# Massive and efficient encapsulation of single cells in monodisperse droplets and collagen–alginate microgels using a microfluidic device

**DOI:** 10.3389/fbioe.2023.1281375

**Published:** 2023-11-15

**Authors:** Dan Liu, Tingting Xuanyuan, Xufang Liu, Wenzhu Fu, Wenming Liu

**Affiliations:** Departments of Biomedical Engineering and Pathology, School of Basic Medical Science, Central South University, Changsha, Hunan, China

**Keywords:** droplet microfluidics, single cell, microgel, biomimetic microenvironment, high-throughput manipulation

## Abstract

Single-cell manipulation is the key foundation of life exploration at individual cell resolution. Constructing easy-to-use, high-throughput, and biomimetic manipulative tools for efficient single-cell operation is quite necessary. In this study, a facile and efficient encapsulation of single cells relying on the massive and controllable production of droplets and collagen–alginate microgels using a microfluidic device is presented. High monodispersity and geometric homogeneity of both droplet and microgel generation were experimentally demonstrated based on the well-investigated microfluidic fabricating procedure. The reliability of the microfluidic platform for controllable, high-throughput, and improved single-cell encapsulation in monodisperse droplets and microgels was also confirmed. A single-cell encapsulation rate of up to 33.6% was achieved based on the established microfluidic operation. The introduction of stromal material in droplets/microgels for encapsulation provided single cells an *in vivo* simulated microenvironment. The single-cell operation achievement offers a methodological approach for developing simple and miniaturized devices to perform single-cell manipulation and analysis in a high-throughput and microenvironment-biomimetic manner. We believe that it holds great potential for applications in precision medicine, cell microengineering, drug discovery, and biosensing.

## Graphical Abstract

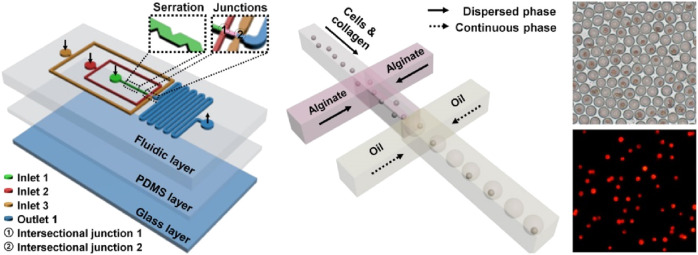

## 1 Introduction

Single-cell manipulation is of great interest in diverse analytical applications in life science for investigating individual cells at the genomic, proteomic, and phenomic levels and exploring single-cell identity and heterogeneity involving mitosis/proliferation, metabolism, drug resistance, and so on (Zhang et al., 2020). Classic methods of single-cell operation such as fluorescence-activated flow cytometry, microscopic manipulation, and laser microdissection have been utilized over decades and have presented great significance in unraveling biological complexity (Ramser and Hanstorp, 2010; Pekle et al., 2019; Läubli et al., 2021; Zhang et al., 2021). However, these methods rely largely on expensive instruments and have non-popularized protocols of single-cell manipulation. Furthermore, microscopic manipulation and laser microdissection techniques often suffer from the low flux of single-cell operation. To accelerate the widespread implementation of micromanipulation in popular single-cell analysis and projects, simple and user-friendly methods for high-throughput single-cell handling are required.

Microfluidics, as an emerging micromanipulation technology in this century, has shown remarkable performance in the spatiotemporal manipulation of fluids and samples, including various types of cells, in a microscale manner (Chen et al., 2020; Liu X. Y. et al., 2021a; Xing et al., 2022). The development and application of single-cell platforms is one of the research hot spots in the microfluidic field (Chung et al., 2019; Anggraini et al., 2022). Over the past 20 years, a considerable number of microfluidic devices have been fabricated, tested, and used for cell manipulation (e.g., trap/capture, isolation, and localization) based on either passive (i.e., mechanical/hydrodynamic) (Liu et al., 2012; Yun et al., 2013) or active force control (i.e., magnetic, electrical, and acoustic) (Ding et al., 2012; Felton et al., 2012; Anand et al., 2015). Notably, passive trapping by microdroplets/structures (e.g., wells and gaps) and active operation by acoustic tweezers allow microscale single-cell control in time and space (Hosic et al., 2016). The capability of massive cell manipulation allows for their applications in the throughput identification of cellular composition and activities (e.g., cell differentiation and gene expression) at single-cell resolution. For instance, the capture of individual cells using a customized microwell array can be conveniently applied to study cell adhesion/proliferation, drug resistance, and intracellular enzyme activity in a microfluidic device (Li et al., 2017; Wang et al., 2019). Currently, microdroplet devices seem to be the most favorable microfluidic tools for scholars to conduct single-cell responses and omics investigations (Klein et al., 2015; Salmen et al., 2022). The independence of monodisperse single-cell droplets effectively prevents cross-contamination and mutual interference between each pair of samples (Salomon et al., 2019; Liu et al., 2020), which is not the case with single-cell arrays in microwell devices. High-frequency (generally from 10 to 1,000 per second) droplet generation in the device gives rise to a large number of individual cell encapsulations (Joensson and Svahn, 2012; Lareau et al., 2019; Brower et al., 2020), which is critical for performing genome sequencing and profiling of single cells and is inaccessible to the existing active microfluidic platforms (typically 10 to 100 once) (Felton et al., 2012; Ng et al., 2015; Guo et al., 2016). From the perspective of device fabrication, the droplet microfluidic system usually containing a single fluidic layer makes its fabrication quite simple, being different from either the microwell array-based microfluidic device with multiple layers or the acoustic chip with additional transducers. While fabrication and operation of droplet microfluidics are simple, low single-cell encapsulation in microdroplets is still one of the key problems in most reported passive microfluidic devices, which commonly produce a single-cell encapsulation rate of up to 20%–30% (Joensson and Svahn, 2012; Utech et al., 2015). On the other hand, solidification of components in droplets to form micro-hydrogels (microgels) and simulate extracellular microenvironments (stromal matrix like collagen and fibronectin) can provide a more physiological microenvironment in three dimensions for *in vitro* cell manipulation and analysis (Kim et al., 2011; Zheng et al., 2019). Single-cell microgel production in various types of materials, such as polyethylene glycol, sodium alginate, agarose, hyaluronic acid, and gelatin, has been proposed by global researchers (Zhang et al., 2012; Rakszewska et al., 2016; Li et al., 2018; Leonaviciene et al., 2020; Wong et al., 2020). The hydrogel materials are characterized by high biocompatibility and a three-dimensional (3D) porous framework; however, most of them do not belong to the *in vivo*-derived stromal material family. Therefore, a high-throughput, easy-to-operate, and microenvironment-simulated methodology of single-cell encapsulation depending on passive force control for enhanced cell encapsulation and biomimetic purpose has remained largely out of reach in the droplet microfluidic device with simple fabrication.

In this study, we describe a facile and massive single-cell encapsulation in microenvironment-biomimetic microdroplets and microgels using a simple-to-fabricate/use microfluidic device. A polydimethylsiloxane (PDMS) microfluidic device with a single functional layer containing the flow-focusing structure and cell-scattering channel was established. Microfluidic generation of both droplets and microgels was evaluated methodically at different perfusions. We verified that the device was able to abundantly fabricate monodisperse droplets/microgels of uniform geometry at microscale control. Next, multi-group encapsulation tests were conducted in the device under various loading conditions to explore single-cell encapsulation. The single-cell droplets and microgels with biomimetic features were successfully and efficiently fabricated by microfluidic and off-chip operations using human breast adenocarcinoma cells and collagen/alginate materials. Microscale droplet/microgel manipulation was monitored dynamically using real-time imaging and assessed quantitatively via mathematical statistics.

## 2 Materials and methods

### 2.1 Device design and fabrication

The droplet microfluidic device consisted of a functionally fluidic layer (channel: 40–200 µm in width and 18 µm in height) and a thin PDMS layer on a glass slide. The fluidic layer contained a channel network for sample loading, droplet generation, and cell encapsulation. Three inlets, set up from the inside to the outside of the fluidic layer, were designed for the introduction of hydrophilic (inlets 1 and 2) and hydrophobic (inlet 3) samples. One outlet was set for fluidic discharge.

The device was fabricated using soft lithography (Liu W. M. et al., 2021b). In brief, a mold for the fluidic layer was fabricated by ultraviolet patterning of SU-8 photoresist (18 µm in thickness, MicroChem, United States) on a silicon wafer (Kaihua Shunchen Electronic Technology Ltd., China). Second, a degassed PDMS mixture (RTV 615 A: B = 10 : 1, Momentive Performance Materials, USA) was poured onto the mold for obtaining a 5-mm-thick layer. After baking at 80°C for 3 h, the fluidic layer was peeled off from the mold. Inlet/outlet holes were punched using a hole puncher. The fluidic layer was trimmed using a blade, cleaned using a Scotch tape, and then assembled onto the glass slide. The glass was pre-coated (2,000 rpm, 75 s) with the PDMS mixture and baked at 80°C for 8 min before the layer assembly. The device was ready for use after baking at 80°C for 48 h.

### 2.2 Cell culture

Human breast adenocarcinoma (MCF-7) cells were obtained from the Chinese Academy of Sciences (Shanghai, China). Cells were routinely cultured using Dulbecco’s modified Eagle’s medium (DMEM, Gibco) supplemented with 10% fetal bovine serum (FBS, Gibco), 100 units/mL penicillin, and 100 μg/mL streptomycin under a humidified atmosphere with 5% CO_2_ at 37°C. To acquire cell suspension, cells were harvested using 0.25% trypsin treatment, centrifuged at 1,200 rpm for 3 min, and then re-suspended in freshly supplemented DMEM. The cell density in the suspension was measured using a hemocytometer.

### 2.3 Microfluidic droplet/microgel generation and cell encapsulation

The device was first sterilized using UV lighting for 2 h, followed by rinsing with autoclaved phosphate-buffered saline (PBS, 0.01 M, pH 7.4). An aqueous solution containing Pluronic F127 solution (20 mg/mL, Sigma-Aldrich) was introduced into the device for channel modification. To generate microdroplets and microgels, ultra-purified water and sodium alginate (30 mg/mL in water, Hyzlin Biology Development Co., Ltd., Qingdao, China) were used as the aqueous phase (dispersed phase) and introduced from inlets 1 and 2 into the device, respectively. Calcified oleic acid was used as the oil phase (continuous phase) and introduced from inlet 3 into the device. The flow rate for sample introduction ranged from 10 to 350 μL/h and was controlled by syringe pumps (longer pump). The ratio of the flow rate of inlet 1 to that of inlet 2 was 1:2. The droplets were formed naturally at the intersectional junction region between dispersed phase and continuous phase flows. To obtain microgels, the generated droplets were collected in a 2% CaCl_2_ bath, which was used to enhance gelation. The alginate-Ca microgels were then produced after gelation for 10 min.

For cell encapsulation in microdroplets and microgels, MCF-7 cells with different cell densities (6–15 × 10^6^ cells/mL) in DMEM containing the neutralized collagen-I (20 mg/mL, Beijing Solarbio Science & Technology Co., Ltd., China) were introduced from inlet 1 into the device. Sodium alginate (30 mg/mL in DMEM) was loaded from inlet 2 into the device. The oil-phase flow was introduced from inlet 3 into the channel at the same time. Cells were trapped in the formed droplets, and single-cell droplets were generated. Furthermore, for cell encapsulation in microgels, the cell-loaded droplets were collected in DMEM containing 2% CaCl_2_ and incubated for 10 min to enhance gelation. The single-cell microgels were centrifuged at 500 rpm for 5 min and finally dispersed in fresh DMEM. The flow rates of dispersed and continuous phases were controlled as mentioned previously.

### 2.4 Cell staining

To visualize and distinguish single cells in droplets, fluorescence labeling of cells was performed before microfluidic cell encapsulation. Dil (i.e., 1,1′-dioctadecyl-3,3,3′,3′-tetramethylindocarbocyanine perchlorate, 10 μM in DMEM; Sigma-Aldrich), a lipophilic fluorescent dye, was used to stain the membrane of MCF-7 cells. The staining process was carried out at 37°C for 10 min. The labeled cells were then centrifuged (1,200 rpm for 3 min) and re-suspended in freshly supplemented DMEM. To assess the viability of cell samples, double fluorescence staining was conducted using fluorescein diacetate (FDA, Sigma-Aldrich) and propidium iodide (PI, Sigma-Aldrich). Cell samples were incubated in an FDA/PI (10 μg/mL for each in DMEM) solution at 37°C for 10 min, followed by a PBS rinse.

### 2.5 Microscopy and image analysis

An inverted microscope (Olympus, CKX53) with a charge-coupled device camera (Olympus, DP74) and a mercury lamp (Olympus, USH-103OL) was utilized for acquiring optical/fluorescent pictures. A stereomicroscope (Olympus, SZX10) with a complementary metal oxide semiconductor camera (FluoCa, BioHD-C20) was used to obtain microfluidic videos. The topological morphology of fluidic components in the device was characterized using a scanning electron microscope (SEM, Hitachi, s-3400N). Image analysis and statistical evaluation (e.g., the roundness of microgels) were carried out using Image-Pro Plus 6.0 (Media Cybernetics, Silver Spring, MD) and SPSS 12.0 (SPSS Inc.) software programs, respectively. All experimental data were shown as means ± standard deviations. All plots in figures were prepared using OriginPro 9.0 software (OriginLab, United States).

## 3 Results and discussion

### 3.1 Device configuration and operation

**FIGURE 1:**
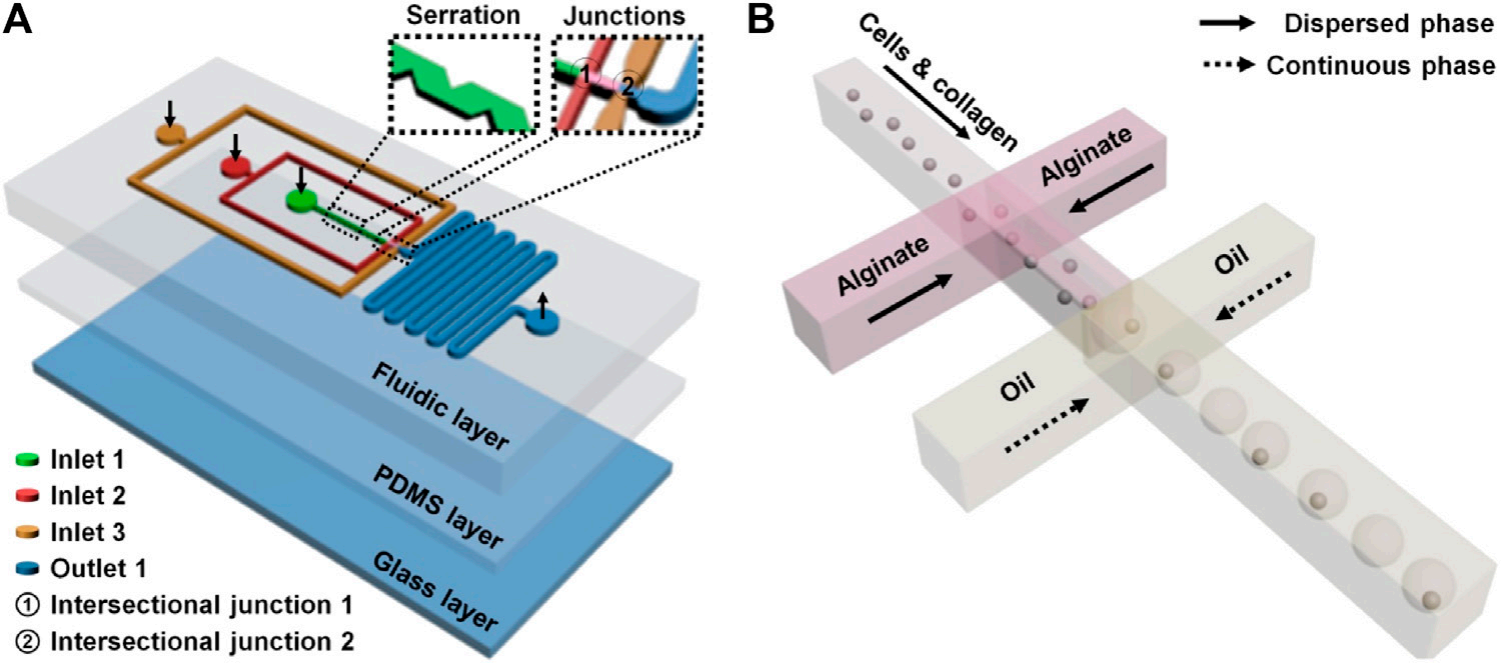
Microfluidic single-cell encapsulation in droplets and collagen–alginate microgels. The microfluidic device **(A)** contained a functionally fluidic layer and two supporting layers (a thin PDMS layer and a glass layer). Three channels (inner, middle, and outmost channels) between inlets (inlets 1, 2, and 3) and the intersectional junctions (junctions 1 and 2) were set for respective sample introductions **(B)** to make single-cell encapsulation. The inner channel with serrate structures from inlet 1 to junction 1 was used to scatter single cells before the encapsulation. The droplets formed at junction 2 flowed to the serpentine channel and were then discharged.

A droplet microfluidic device has been recognized as an excellent microplatform for high-throughput manipulation and analysis of biological samples (Liu H. R. et al., 2021c). Droplet microfluidics-based single-cell operation is currently one of the research hot spots due to the desirability of unveiling the inhomogeneity between cells in numerous cellular and molecular behaviors (Mazutis et al., 2013). In this study, the droplet microfluidic device was composed of two functional domains from the inlet side to the outlet side: sample loading and droplet formation domains (Figure 1; Supplementary Figure S1). The sample loading domains included three channels (inner, middle, and outmost channels) between inlets (inlets 1, 2, and 3) and their intersectional junctions (junctions 1 and 2). The inner channel with serrate structures (channel width: 40–80 µm) from inlet 1 to junction 1 was set for enhancing single-cell dispersion. The inner and middle channels, starting from inlets 1 and 2, were intersected at junction 1 for cell/collagen and alginate introduction (i.e., dispersed phase), respectively, when doing the cell encapsulation experiment. The outmost channel from inlet 3 to junction 2 was for loading oil as a continuous phase. The droplet formation domain contained junction 2, which was subsequently connected with a serpentine channel. A flow-focusing structure (i.e., junction 2) was set in the device for droplet formation and cell encapsulation. Dispersed and continuous phase flows encountered at junction 2 and two counter flows of the continuous phase squeezed the forefront fluid of the dispersed phase to form microdroplets based on the precise control of dual-phase flows. Individual cells in the dispersed phase can be encapsulated in droplets naturally. The generated droplets flowed to the serpentine channel and were then discharged at the outlet. The designed and fabricated flow-focusing device was applied to perform a robust, massive, and geometry-homogeneous production of microdroplets with or without single cells.

### 3.2 Microfluidic generation of monodisperse droplets

**FIGURE 2:**
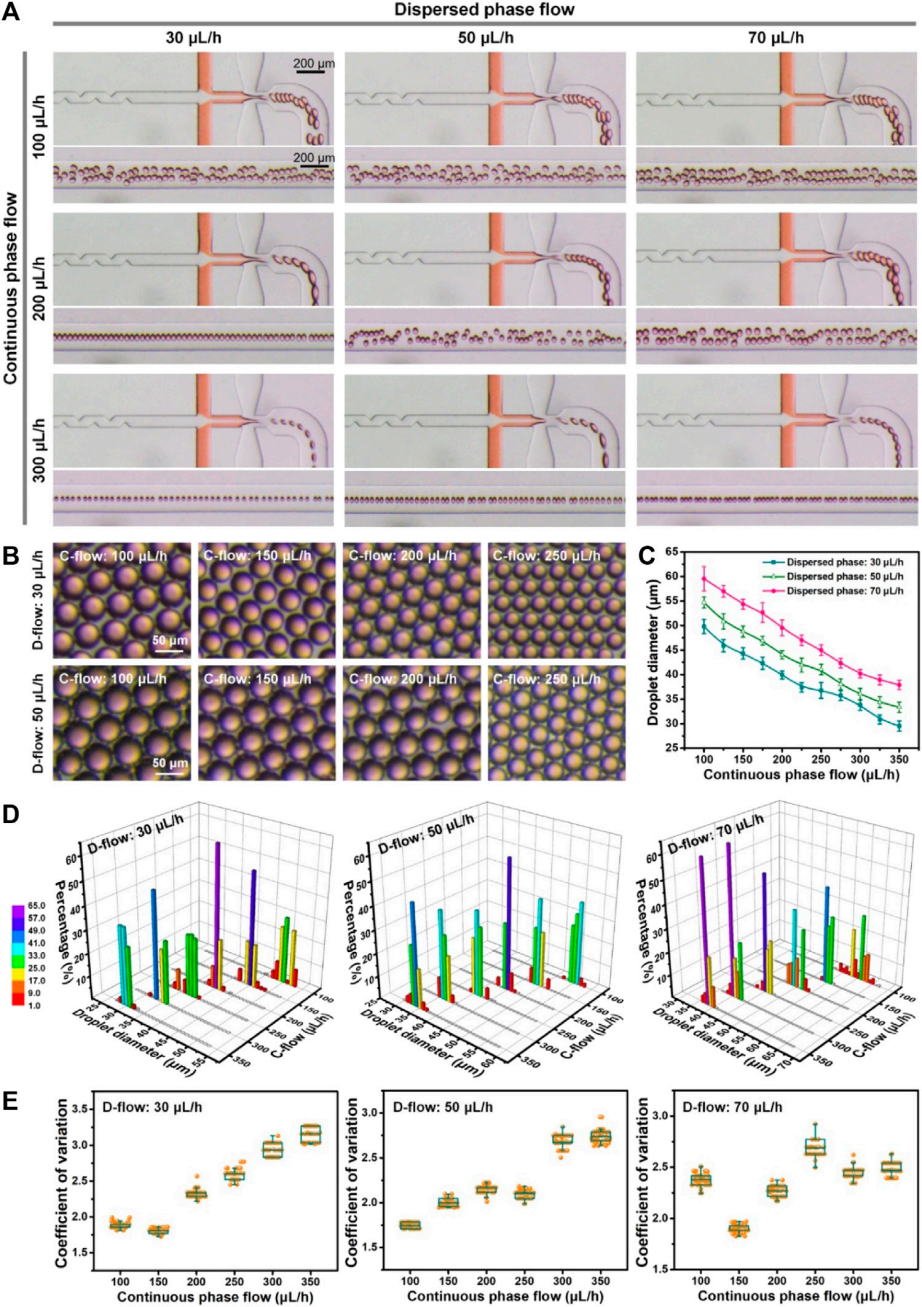
Microfluidic generation of monodisperse droplets at different flow rates of the dispersed phase (30, 50, and 70 μL/h, D-flow) and continuous phase (100–350 μL/h, C-flow). **(A)** Optical images of droplet generation under various flow conditions. Orange food dye was applied for the visualization of flow from the middle channel. **(B)** Optical images of droplets generated under various flow conditions. **(C)** Size of droplets generated at different perfusions. **(D)** Size distribution of droplets fabricated under various flow conditions. **(E)** Coefficient of variation of droplets produced at different flow conditions.

The controllability and stability of monodisperse droplet generation in the flow-focusing device were evaluated initially. The flow condition is recognized as the key factor for the generation and size regulation of droplets. Different flow conditions including the dispersed phase flow (D-flow: 30, 50, and 70 μL/h) and the continuous phase flow (C-flow: 100–350 μL/h) were tested here. The flow rates of fluids at inlets 1 and 2 were specifically set at a ratio of 1:2, which led to a nearly equivalent distribution of flows from both inner and middle channels before arriving at junction 2 (Supplementary Figure S2). The optical and quantified results are shown in Figure 2. It was clear that droplets were stably generated in the device (Figure 2A; Supplementary Figure S2; Supplementary Movies S1–S3). The size of droplets was positively related to the flow rate of the dispersed phase and negatively to the flow rate of the continuous phase (Figures 2B, C). The size (diameter) of the produced droplets ranged from 29.5 ± 1.0 µm to 59.5 ± 2.5 µm. The biggest droplets were generated at the maximum dispersed phase flow (70 μL/h) and minimum continuous phase flow (100 μL/h). On the contrary, the smallest droplets were produced at the minimum dispersed phase flow (30 μL/h) and maximum continuous phase flow (350 μL/h). It was suggested that the size of the generated droplets can be controlled by intentionally regulating flow conditions in both continuous and dispersed phases. Additionally, the generation rate of monodisperse droplets ranged from tens to hundreds of droplets per second, which supports the capability of the microfluidic device for massive droplet production (Salomon et al., 2019; Brower et al., 2020).

Next, the size distribution of droplets produced under various flow conditions was assessed quantitatively (Figure 2D). It was shown that the percentage of droplets with the specific diameter ±3.0 µm was over 98% and up to 100% under most flow controls of continuous and dispersed phases, except one flow condition (i.e., dispersed phase: 70 μL/h; continuous phase: 100 μL/h), which achieved only up to 86%. The results verified that the device could generate droplets with high size uniformity, relying on precise flow control. Furthermore, the quantified data (Figures 2E) showed that the coefficient of variation (CV) of each droplet generation was less than 3.2, and the minimum CV could reach 1.7, which is similar to previous achievements (Nisisako and Torii, 2008). This indicated the preeminent monodispersity of fabricated droplets in the microfluidic device. Totally, a high degree of controllability and monodispersity of microfluidic droplet generation was demonstrated experimentally using the established microfluidic device, which can largely strengthen the subsequent microfluidic droplet manipulation for microgel production and single-cell encapsulation. The microfluidic protocols of highly monodisperse droplet production could be selectively applied for the following experiment.

### 3.3 Controllable production of microgels

**FIGURE 3:**
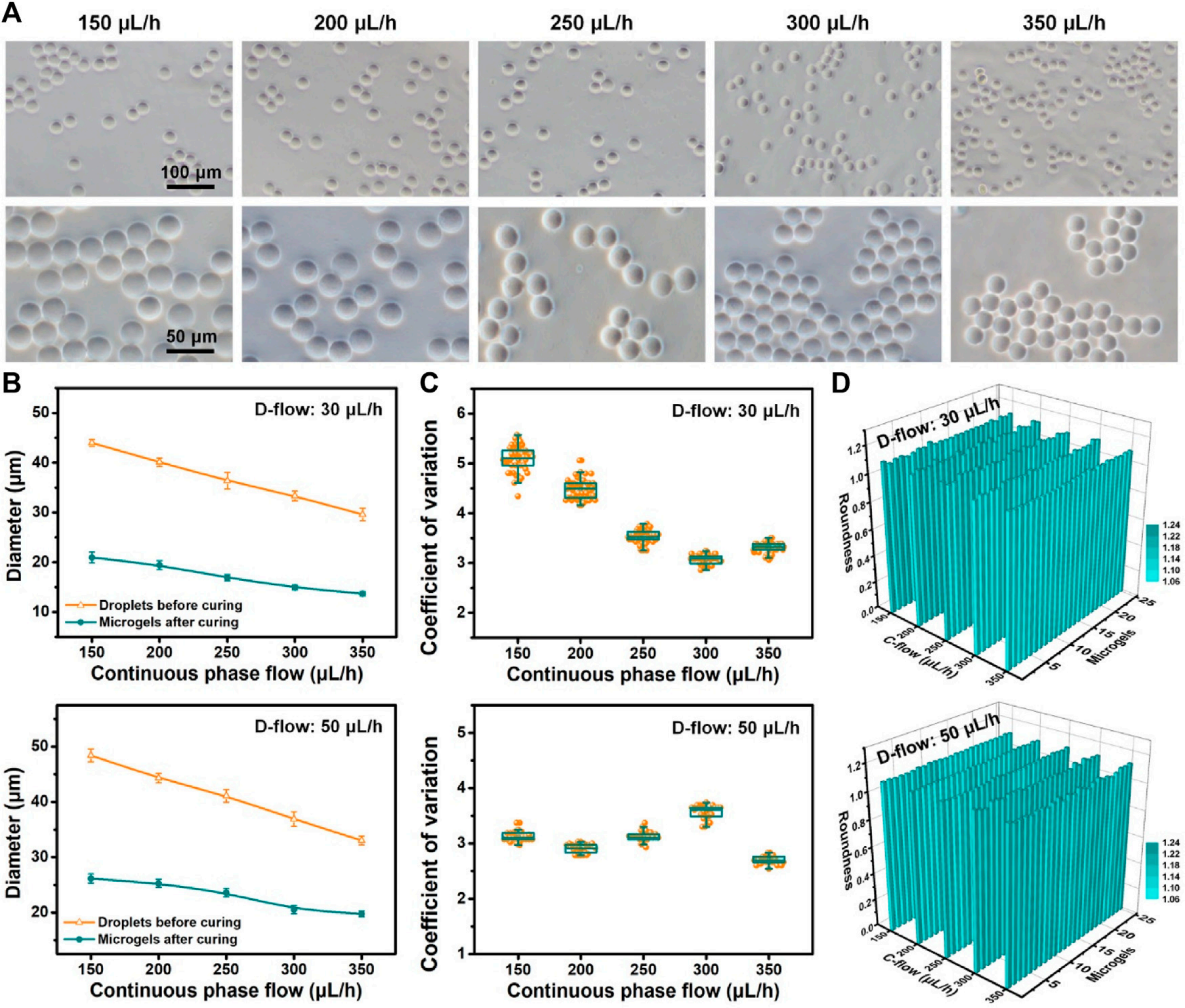
Controllable production of microgels. **(A)** Optical images of alginate microgels produced based on the microfluidic operation with flow control (dispersed phase: 50 μL/h; continuous phase: 150–350 μL/h). **(B)** Size of droplets before curing and microgels after curing produced under different D-flow (top: 30 μL/h; bottom: 50 μL/h) and C-flow conditions. **(C, D)** Coefficient of variation **(C)** and roundness **(D)** of microgels produced under different D-flow (top: 30 μL/h; bottom: 50 μL/h) and C-flow conditions.

To construct a 3D microenvironment for single-cell manipulation/analysis, controllable and mass production of hydrogel particles (microgels) was necessary (Utech et al., 2015; Wong et al., 2020). In this study, sodium alginate, a highly biocompatible material for living cell manipulation in 3D, was used as a component of generated droplets for liquid solidification and microgel formation. The gelation of alginate-loaded droplets is based on the crosslinking caused by the chelation of calcium ions and carboxylic acid groups in the glucose unit of sodium alginate (Liu et al., 2022). So this gelation process is quite fast. After the formation of droplets in the flow-focusing device, they were collected in a solution containing calcium chloride for sufficient gelation. We tested multi-group productions of microgels with different sizes via on-chip fluidic control (dispersed phase: 30 and 50 μL/h; continuous phase: 150–350 μL/h) and off-chip gelation (Figure 3). Various sizes of microgels with regular shapes and a clear outline can be fabricated on account of microfluidic size control of droplets. Based on the extensive statistical analysis (Figures 3A, B; Supplementary Figure S3), we found that the diameter of microgels decreased with the increased flow rates of the continuous phase. Increasing the flow rate of the dispersed phase can make the size of microgels bigger. The size of the produced microgels ranged from 13.7 to 26.1 µm in diameter. For each flow condition (e.g., dispersed phase: 50 μL/h; continuous phase: 200 μL/h), we observed that the size of generated droplets (44.3 µm in diameter) before curing was bigger than that of cured microgels (25.3 µm in diameter). The main explanation was the shrinkage property of alginate-Ca micro-hydrogels (Ren et al., 2015). The shrinkage rates of microgels were typically below 55% (e.g., 50 μL/h D-flow: 46.0% and 40.1% at 150 and 350 μL/h C-flow conditions, respectively). In addition, the size of either droplets or microgels from repeatable tests was similar, suggesting a stable microfluidic production. Furthermore, the quantitative results (Figure 3C) showed that the CV values of microgel fabrication corresponding to the aforementioned flow conditions ranged from 2.7 to 5.1. This suggested that the high monodispersity of fabricated microgels was achieved. We found that a higher dispersed phase flow (50 μL/h) seemed to broadly make the CV value smaller, meaning a higher monodisperse microgel production.

The geometry distribution of these microgels was evaluated as follows. The quantitative results (Figure 3D) showed that the roundness values of microgels generated under diverse flow conditions of dispersed and continuous phases were totally close to 1.1, which suggested that the produced microgels were nearly circular and exhibited geometry that was homogeneous between themselves. These results demonstrated that the controllable production of microgels with high monodispersity and high uniform geometry was accomplished successfully. Based on the aforementioned high-throughput microfluidic droplet generation, mass fabrication of microgels can also be realized.

### 3.4 Single-cell encapsulation in biomimetic droplets and microgels

**FIGURE 4:**
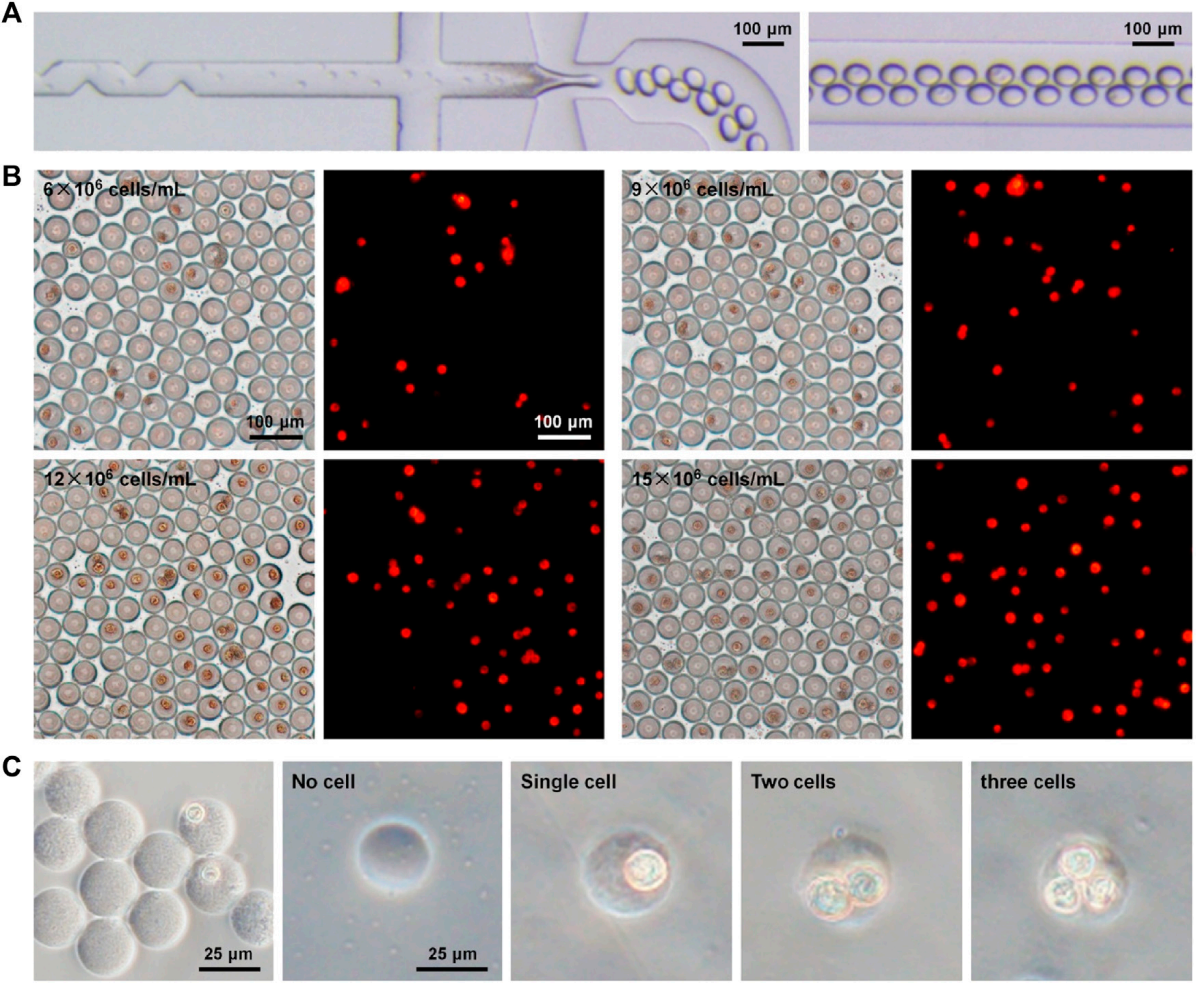
Single-cell encapsulation in biomimetic droplets and microgels. **(A)** Optical images of cell encapsulation in collagen–alginate droplets in the microfluidic device. **(B)** Optical and fluorescent images of droplets after microfluidic cell encapsulation at different cell densities (6–15 × 10^6^ cells/mL). Cells were pre-labeled using fluorescent Dil (red) staining. **(C)** Cell-encapsulated microgels in collagen–alginate.

Depending on the robust and controllable production of microdroplets and microgels, we further performed the experimental investigation of single-cell encapsulation using the established microfluidic operation. Following the aforementioned flow control, a culture medium containing MCF-7 cells and collagen-I (the major component of the extracellular matrix *in vivo*) was loaded from inlet 1 into the inner channel, and sodium alginate in the medium was introduced from inlet 2 into the middle channel. Both of them were in the dispersed phase, and the oil flow in the outmost channel was in the continuous phase. A 50 μL/h dispersed phase flow and a 150 μL/h continuous phase flow were used in this part of the study. Due to passive cell manipulation in the flow-focusing device, the number of loaded cells in every droplet is, to a large extent, dominated by Poisson distribution, which is why the single-cell encapsulation rate is low using passive devices (Collins et al., 2015; Matula et al., 2020). To improve single-cell encapsulation, we tested microfluidic droplet generation and cell encapsulation at four different densities (6–15 × 10^6^ cells/mL) in the device with a channel containing serrate structure. These structures were specifically set in the inner channel to influence the flow field (Yun et al., 2013), which scattered cells during the loading process that can be confirmed by resultant images (Figure 4A; Supplementary Figure S4; Supplementary Movie S4). It was not apparent that the addition of these serrate structures negatively disturbed the fluid flow during cell loading. For fluorescent visualization, MCF-7 cells were pre-labeled using Dil (red) staining. The optical and fluorescent images of cell encapsulation in droplets are shown in Figure 4B. It was observed that a large quantity of cell encapsulation in droplets was completed based on the established high-throughput droplet formation (hundreds of droplets per second) using the microfluidic device. Several droplets containing cells were fabricated while using a higher density of loaded cells. The single-cell encapsulation in droplets increased quantitatively with the introduction of more cells. Moreover, droplets were cured in the calcium bath to form cell/collagen-laden microgels (Figure 4C). We noticed that these stroma-biomimetic particles had intact structure and uniform morphology. The number of cells in gels corresponded to the efficiency of cell encapsulation in droplets.

**FIGURE 5:**
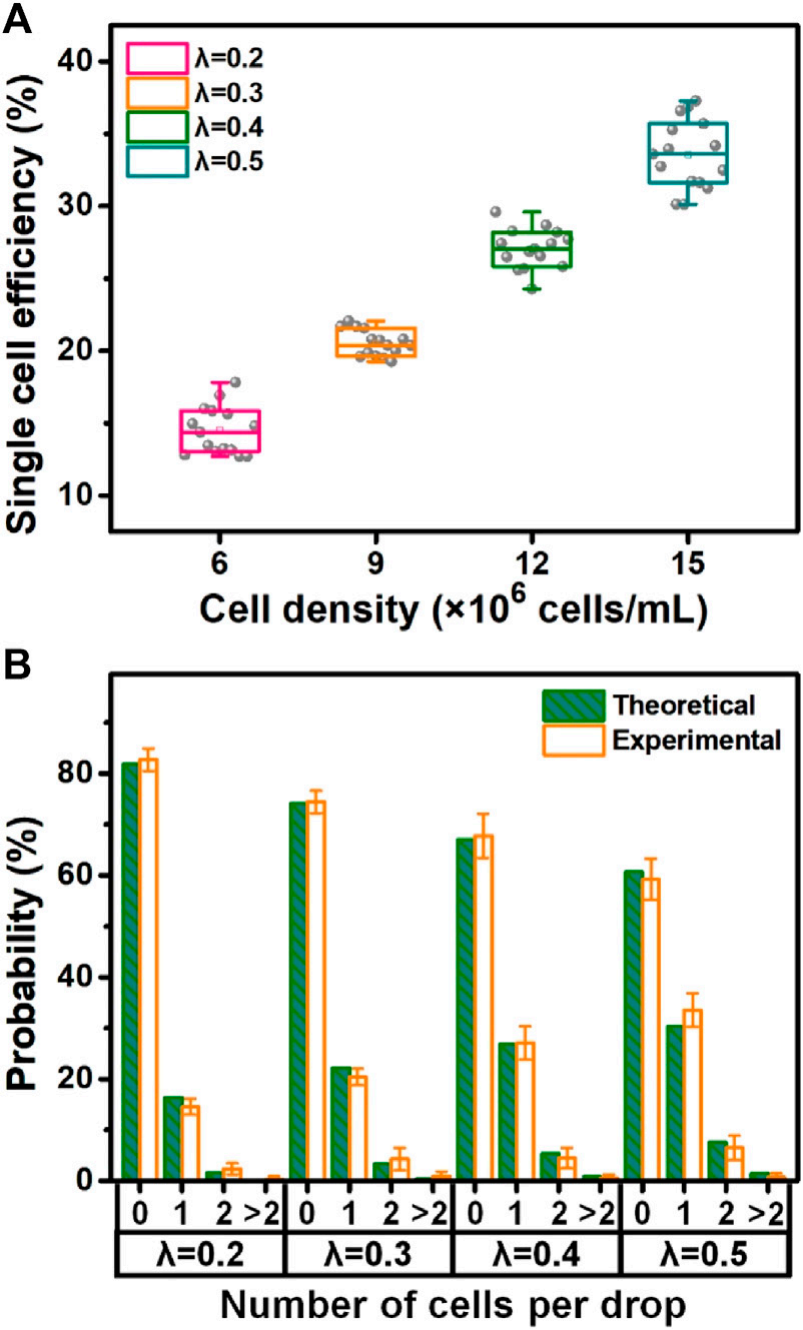
Quantitative evaluation of microfluidic single-cell encapsulation. **(A)** Single-cell encapsulation rate at different densities of cell loading. **(B)** Percentage of theoretical and experimental cell encapsulations (i.e., non-cell, single-cell, two-cell, and over two-cell encapsulations) under various loading conditions (i.e., λ = 0.2 vs. 6 × 10^6^ cells/mL; λ = 0.3 vs. 9 × 10^6^ cells/mL; λ = 0.4 vs. 12 × 10^6^ cells/mL; and λ = 0.5 vs. 15 × 10^6^ cells/mL).

The cell encapsulation was quantitatively assessed based on the image analysis (Figure 5). The results (Figure 5A) showed that the encapsulation rates of single cells during microfluidic operations at different densities (6, 9, 12, and 15 × 10^6^ cells/mL) were 14.6%, 20.4%, 27.1%, and 33.6%, respectively. On account of Poisson distribution, *λ* as the average number of cells per droplet volume was applied for appropriately matching four densities of loaded cells (i.e., *λ* = 0.2 vs. 6 × 10^6^ cells/mL; *λ* = 0.3 vs. 9 × 10^6^ cells/mL; *λ* = 0.4 vs. 12 × 10^6^ cells/mL; and *λ* = 0.5 vs. 15 × 10^6^ cells/mL). We compared the experimental data to the theoretical values of cell encapsulation and found that there was a high coincidence between two data sources under the same microfluidic conditions. It was verified that cell encapsulation, especially single-cell encapsulation in monodisperse droplets and collagen–alginate microgels, was positively associated with the *λ* value and cell density. Surprisingly, the results showed that the efficiency of actual single-cell encapsulation was higher than that (30.3%) from Poisson statistics, while *λ* is 0.5 corresponding to the cell density of 15 × 10^6^ cells/mL (Figure 5B). This phenomenon was mainly attributable to the specific design of the inner channel of the device for adequate cell dispersion to fit the droplet generation dynamics. The achievement of single-cell encapsulation was not the case for most of the reported passive droplet microfluidic systems (Headen et al., 2018; Zhang et al., 2019). The introduction of the stromal composition into microgels for building an *in vivo*-like extracellular microenvironment allows diverse biomimetic analyses of single-cell events, including proliferation, migration, protein/gene expression, and drug resistance. High cell viability (over 90%), being similar to other achievements (Utech et al., 2015; Salomon et al., 2019), can be maintained during these microfluidic single-cell encapsulation tests (Supplementary Figure S5). This approach confirmed that the microfluidic device in this study was able to conduct massive and efficient single-cell encapsulation in monodisperse droplets and stroma-biomimetic microgels. In addition, cell-free droplet/microgel removing and single-cell droplet/microgel purification are quite beneficial for improving their biomedical applications, which will be involved in our future studies.

## 4 Conclusion

In conclusion, we developed a simple, massive, and efficient methodology of single-cell encapsulation based on high-throughput generation of biomimetic droplets and micro-hydrogels with high monodispersity and geometric homogeneity using an easy-to-fabricate/operate microfluidic device. The high controllability of droplet and microgel generation with uniform geometry was demonstrated by a systematic investigation of the fluidic impact on this fabricating process. The microfluidic device with a flow-focusing structure enabled the production of hundreds of droplets and microgels per second. Furthermore, single-cell encapsulation in monodisperse droplets and microgels was efficiently improved by intentional device design and loading optimization. Collagen–alginate microgels provided single cells in a more biomimetic microenvironment relative to previously fabricated microparticles (Zhang et al., 2012; Rakszewska et al., 2016; Li et al., 2018; Wong et al., 2020). The *in vitro* construction of extracellular stroma conditions at the microscale is quite useful for performing microenvironment-simulated single-cell exploration. We anticipate that the microfluidic achievement may promote the development of facile droplet microfluidic systems to conduct controllable, high-throughput, and efficient manipulation of single cells for life exploration with *in vivo*-like microenvironment rebuilding, which would be potentially valuable in various biomedical fields, including tumor biology, basic medicine, genetics, biomedical engineering, and pharmacology.

## Data Availability

The raw data supporting the conclusion of this article will be made available by the authors, without undue reservation.
