# Supplementary material for: Massive and efficient encapsulation of single cells in monodisperse droplets and collagen–alginate microgels using a microfluidic device
Source: Front Bioeng Biotechnol. 2023 Nov 15;11:1281375. doi: 10.3389/fbioe.2023.1281375 (PMC10684782; doi:10.3389/fbioe.2023.1281375)
Supplement: Supplementary file 4 [file DataSheet1.PDF]

Supporting Information

Massive and efficient encapsulation of single cells in monodisperse droplets and collagen-alginate microgels using a microfluidic device

Dan Liu,[1] Tingting Xuanyuan,[1] Xufang Liu,[1] Wenzhu Fu,[1] Wenming Liu*,[1]

[1]Departments of Biomedical Engineering and Pathology, School of Basic Medical Science, Central South University, Changsha, Hunan 410013, China

*To whom correspondence should be addressed. W. Liu e-mail: liuwenming0229@csu.edu.cn; Phone: +86-731-82650401.

Supplementary Figures

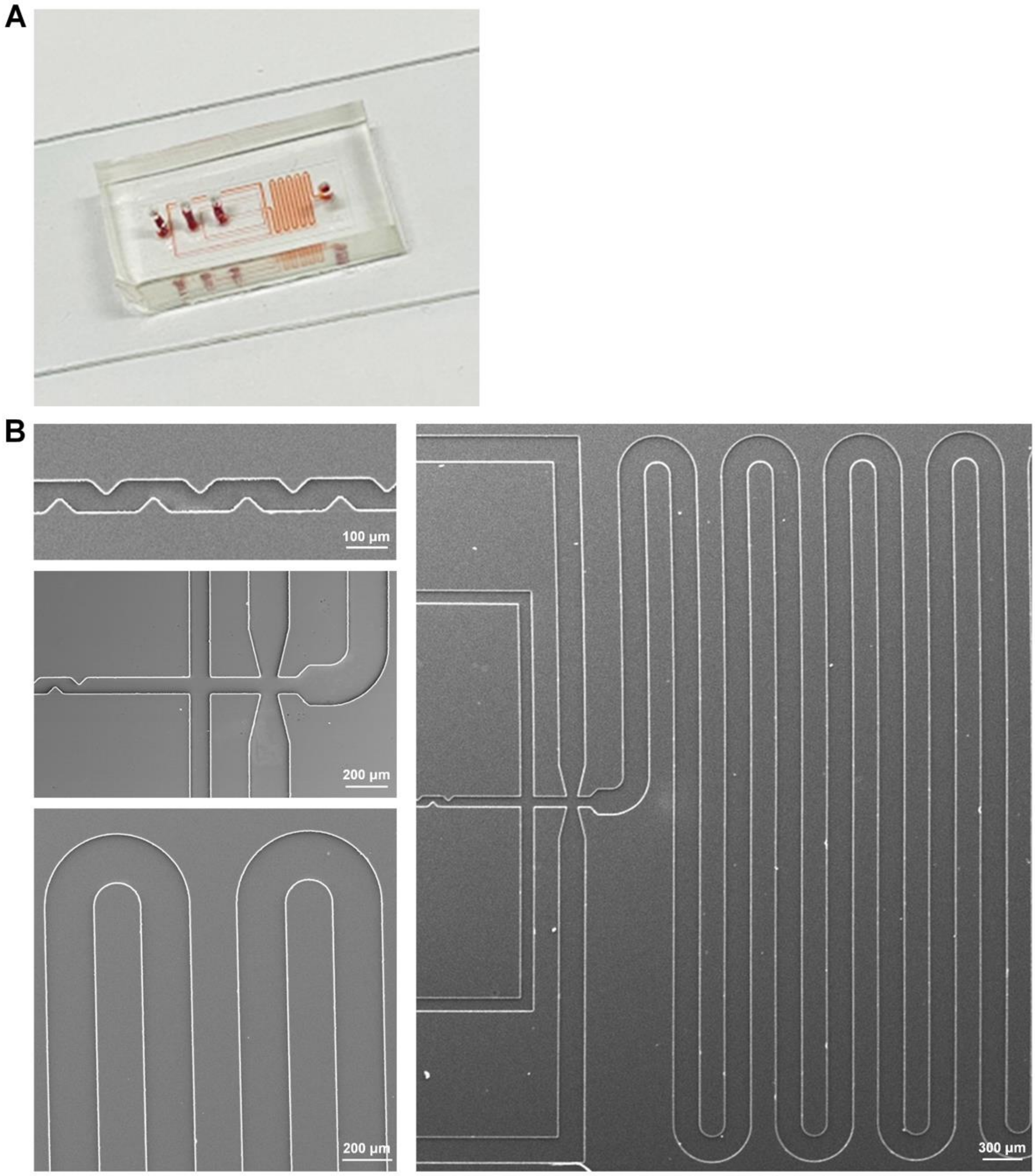

Figure S1. PDMS microfluidic device. (A) The actual PDMS microfluidic device. (B) SEM images of microfluidic components including the inner channel with serrate structures, the middle and outmost channels, intersectional junctions, and the serpentine channel.

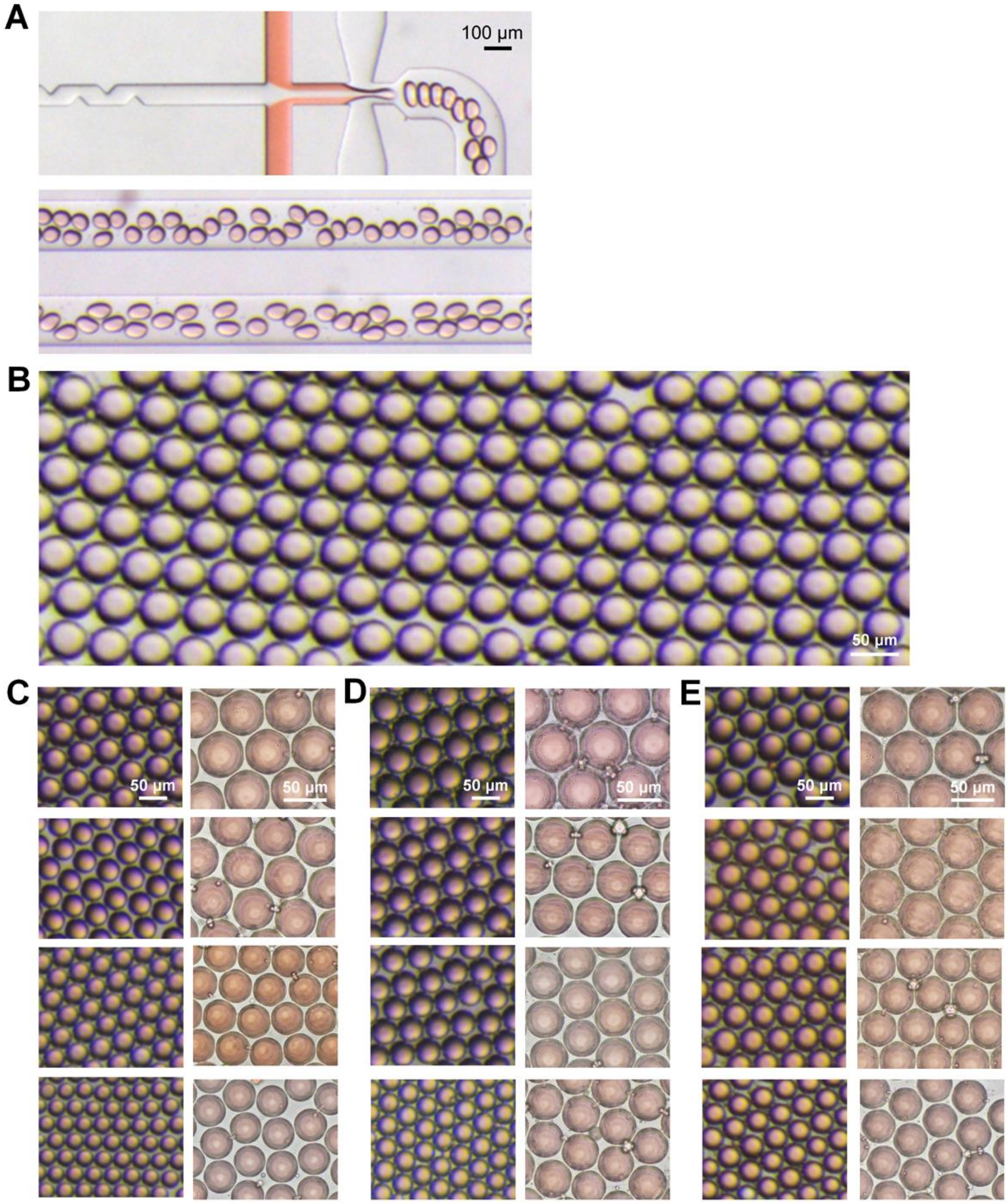

Figure S2. Microfluidic droplet generation at various flow conditions. (A) Optical images of droplet generation at 30 μL/h dispersed phase and 150 μL/h continuous phase flows. (B) Optical image of size-uniform droplets prepared using the microfluidic device. (C) Optical images of droplets generated at a dispersed phase (30 μL/h) and various continuous phase (top to bottom: 100, 150, 200, and 250 μL/h) flow conditions. (D) Optical images of droplets generated at a dispersed phase (50 μL/h) and various continuous phase (top to bottom: 100, 150, 200, and 250 μL/h) flow conditions. (E) Optical images of droplets generated at a dispersed phase (70 μL/h) and various continuous phase (top to bottom: 150, 200, 250, and 300 μL/h) flow conditions.

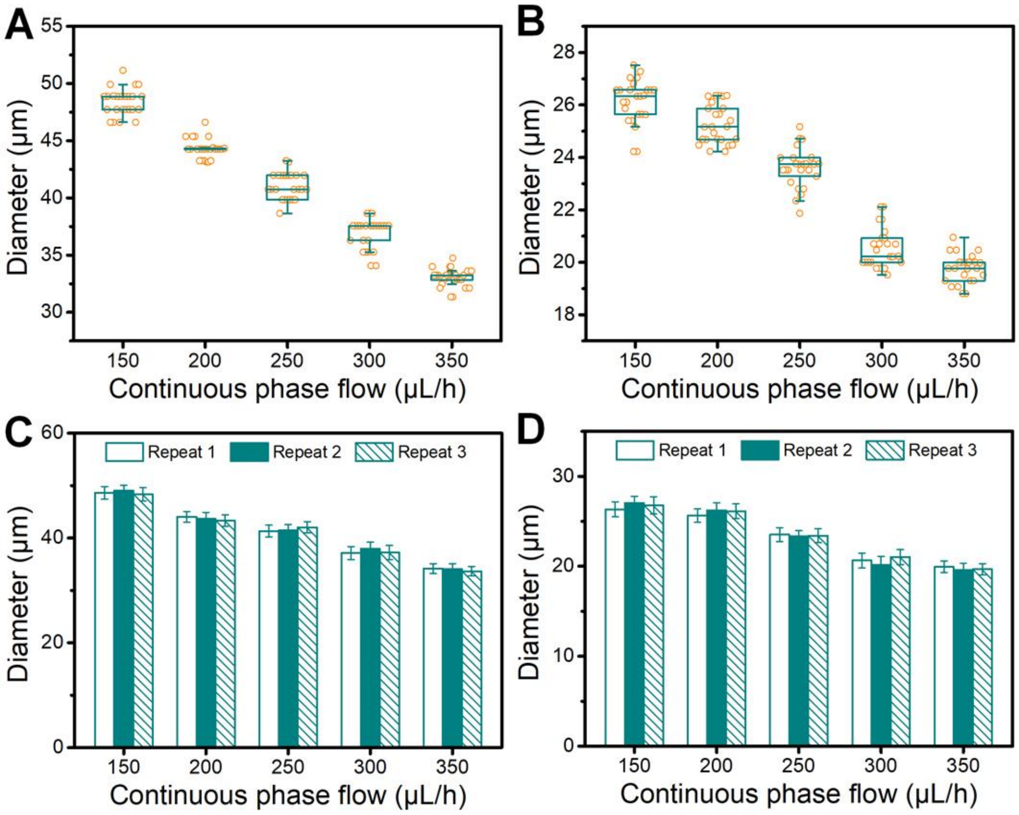

Figure S3. Size of droplets (n=25) before curing (A) and microgels after curing (B) produced at different D-flow (50 μL/h) and C-flow conditions (100 to 350 μL/h). The repeated generation of droplets (C) and microgels (D) were performed thrice. It was shown that the size of droplets or microgels from each generation was similar suggesting a stable and repeatable microfluidic production.

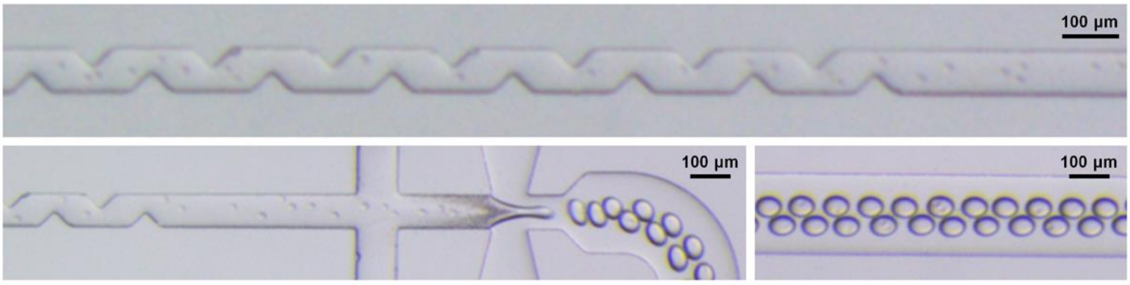

Figure S4. Optical images of cell encapsulation in collagen-alginate droplets in the microfluidic device. It was observed that the loaded cells were well-dispersed in the inner channel containing serrate structures.

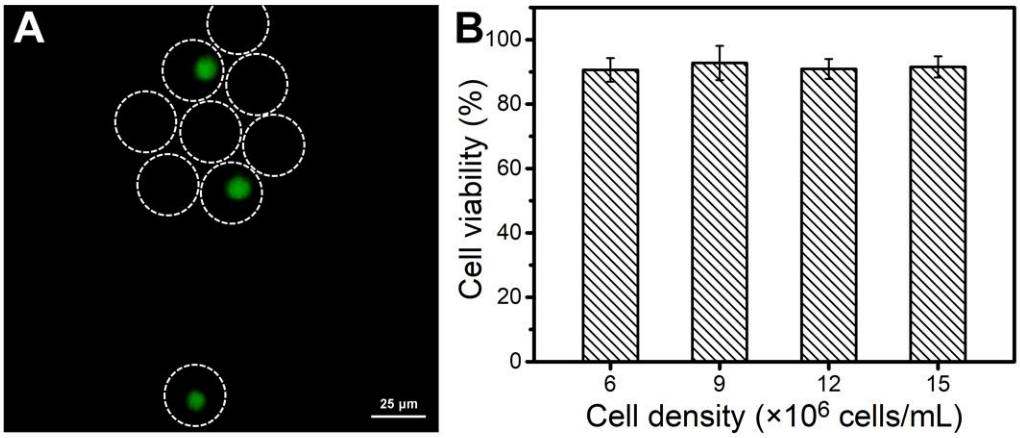

Figure S5. The viability of cells in the microgels. (A) Fluorescent image of single cells in microgels. Live (green) and dead (red) cells were fluorescently labeled using fluorescein diacetate (FDA) and propidium iodide (PI) staining. (B) Quantitative viability analysis of microfluidic cell encapsulation after gelation at different cell densities (6 to 15 $\times 10^6$ cells/mL). It was shown that the percentage of cell viability from each test was over 90%.

Supplementary Movies

Movie S1. Microfluidic generation of monodisperse droplets at 30 μL/h dispersed phase flow and 100 μL/h continuous phase flow.

Movie S2. Microfluidic generation of monodisperse droplets at 50 μL/h dispersed phase flow and 100 μL/h continuous phase flow.

Movie S3. Microfluidic generation of monodisperse droplets at 30 μL/h dispersed phase flow and 250 μL/h continuous phase flow..

Movie S4. Microfluidic cell encapsulation in monodisperse droplets at 50 μL/h dispersed phase flow and 150 μL/h continuous phase flow.
